# A Discovery Rivaling Dr. Jenner's

**Published:** 1882-01

**Authors:** 


### ARTICLE VII.

### *A Discovery Rivaling Dr. Jenner's.*

One of the most important papers read at the meeting of the International Medical Congress, held in London, was that of M. Pasteur, the distinguished biologist, on certain discoveries of his which will enable physicians to convert vaccination from being an isolated and empiric precaution into a wide system of treatment, applicable to many kinds of fever of both man and animals. His investigations have resulted not alone in preventatives for four distinct violent diseases, but in a knowledge of a method of preparing a vaccine for preventing fevers and many—if not all—contagious diseases. M. Pasteur, in his address as reported by the London *Times*, gives in detail his discoveries with respect to two diseases—chicken cholera and splenic fever. It will suffice to trace his method of preparing a vaccine to prevent the first. He takes a chicken about to die of the cholera and draws from its veins a small quantity of blood. Under the microscope this blood is seen to be full of small living creatures, which, for want of a better name, we shall call *microbs*—the name employed by M. Pasteur. A drop of this blood is placed in a close glass vessel containing clear strained, recently boiled broth, made from chicken or other flesh. Great precaution is taken to exclude the organic germs floating in the air; in fact the glass neck of the vessel is closed with a plug of cotton, or is drawn out in a lamp flame and hermetically sealed. The glass vessel, or flask, is kept at a temperature of about 55 degrees Fahrenheit. Its contents at first become turbid from the growth of the microbes nourished by the broth, but at the end of a couple of days the thickness of the broth disappears because the microbes have ceased to develop and have fallen to the bottom of the flask, and things will remain in this condition for months without either liquid or sediment undergoing any visible change, provided the atmospheric germs are kept excluded. After an interval of a month the flask is shaken to mingle its contents, and a drop from it is placed in a second flask, containing fresh broth. A crop of microbes is produced as before, followed by the some clearing of the liquid and falling of sediment. The interval of a month's waiting is repeated, and a drop from this second flask is employed to produce microbes in a third, and so on until there has been, say, a dozen crops of microbes raised. At the end of the process it will be found that the "cultured microbes," to employ M. Pasteur's language, are innocent, and when introduced into the veins of a healthy chicken, fail to produce cholera, as their uncultured ancestors did, yet, at the same time, they prevent the chicken from catching the cholera. In a word, the cultured microbe can be used to vaccinate, and thus protect the farmer's chickens from one of the worst diseases to which foul flesh is heir. The rationale of the process is that the vigor of the microbes is exhausted by any considerable period of suspended animation, a month or less, and when the process is often repeated the enfeebled microbe loses the virulence of his progenitors. The microbe which causes splenic fever in sheep, &c., differs from that producing cholera in its mode of growth, but may be "cultured" to a state of innocency by a process similar to the one just narrated, the strict exclusion of fresh air being in this case indispensable. It is the want of oxygen which seems to occasion the enfeeblement of the microbe during its recurring periods of suspended life. This method of obtaining the vaccine of splenic fever was no sooner made known than it was extensively employed. France loses $5,000,000 worth of sheep annually from splenic fever. To test his method M. Pasteur had fifty sheep given him for experiment by the government. He vaccinated twenty-five of them with his cultured microbes. A fortnight afterward the whole fifty were inoculated with the ordinary uncultured splenic microbe. The twenty-five vaccinated sheep resisted the infection, the other twentyfive died of splenic fever within fifty hours. Since that M Pasteur has vaccinated 20,000 sheep and large numbers of cattle and horses and with good results.—*Sun.*

